# Comparing a Guideline-Based Mobile Health Intervention Versus Usual Care for High-Risk Adolescents With Asthma: Protocol of a Randomized Controlled Trial

**DOI:** 10.2196/69903

**Published:** 2025-07-25

**Authors:** Tamara T Perry, Jessica H Turner, Ariel Berlinski, Larry A Simmons, Rita H Brown, Kaymon Neal, Sarah A Marshall, Xing He, Simon Chung, Andrew Brown, Horace J Spencer 3rd, Jiang Bian

**Affiliations:** 1 Department of Pediatrics University of Arkansas for Medical Sciences Little Rock, AR United States; 2 Arkansas Children's Research Institute Little Rock, AR United States; 3 Department of Health Behavior & Health Education College of Public Health University of Arkansas for Medical Sciences Little Rock, AR United States; 4 Department of Biostatistics and Health Data Science Indiana University Indianapolis, IN United States; 5 Regenstrief Institute Indianapolis, IN United States; 6 Department of Biostatistics University of Arkansas for Medical Sciences Little Rock, AR United States

**Keywords:** asthma, smartphone application, application, app, mobile health, mHealth, adolescents

## Abstract

**Background:**

Mobile health (mHealth) technology has the ability to integrate personalized health management into patients’ daily routines. In prior investigations of mHealth apps for asthma, patient satisfaction and acceptability have been high. However, rigorous randomized controlled trials (RCTs) examining their effectiveness are sparse; the majority of mHealth asthma apps lack personalization and real-time feedback and fail to include at-risk pediatric populations, and many previous studies are not randomized.

**Objective:**

This full-scale RCT will examine the effectiveness of the Pulmonary Education and Asthma Knowledge Mobile Asthma Action Plan (PEAKmAAP), an interactive mHealth asthma action plan (mAAP) smartphone app, among adolescents compared to enhanced usual care (eUC). The study has 3 aims: (1) examine the effectiveness of PEAKmAAP in reducing asthma morbidity, as measured by the Asthma Control Test (ACT) score, health care use, medication use, and lung function; (2) examine the effectiveness of PEAKmAAP in asthma self-efficacy and medication adherence; and (3) examine the impact of sharing PEAKmAAP-generated data with the primary care provider (PCP) for a subset of enrolled subjects. We hypothesize that the PEAKmAAP groups will experience reduced asthma morbidity compared to the eUC group. Furthermore, we hypothesize that PCP data sharing is expected to enhance PCP prescribing patterns and that more adolescents in the Pulmonary Education and Asthma Knowledge Mobile Asthma Action Plan with data sharing (PEAKmAAP-DS) group will have sustained controlled at follow-up visits compared to PEAKmAAP alone or eUC.

**Methods:**

Using a 3-arm RCT lasting 12 months, we will assess the effectiveness of PEAKmAAP in reducing morbidity among 432 adolescents (age 12-20 years). The study population includes adolescents with uncontrolled symptoms who receive primary care at the Arkansas Children’s Hospital (ACH) or asthma care at ACH specialty clinics. At baseline, participants are randomly assigned to 1 of 3 groups: (1) PEAKmAAP group, (2) PEAKmAAP-DS group, and (3) eUC group using a smartphone app with daily non–asthma-related notifications. Study procedures will include baseline, 3-month, and 12-month in-person visits and telephone visits at 6 and 9 months. In-person visits will measure the ACT score, lung function, and self-efficacy; telephone visits will measure the ACT score. Participants will complete monthly online surveys to assess health care use and medication use.

**Results:**

Recruitment and data collection began in March 2019, and data collection concluded in May 2024. Full data analysis began in December 2024.

**Conclusions:**

This RCT aims to examine the effectiveness of a mAAP with real-time feedback and PCP data sharing. The study addresses existing gaps in knowledge regarding implementation of a mAAP for high-risk adolescents and has the potential to serve as a model for other populations at high risk for asthma.

**Trial Registration:**

ClinicalTrials.gov NCT03842033; https://clinicaltrials.gov/study/NCT03842033

**International Registered Report Identifier (IRRID):**

DERR1-10.2196/69903

## Introduction

Asthma is a major public health concern related to significant childhood morbidity, and despite proven interventions and evidence-based guidelines that have been established for more than 3 decades, 44% of US children have poorly controlled disease [1]. According to the National Asthma Education and Prevention Program’s (NAEPP) guidelines, poorly controlled asthma is defined as having symptoms or rescue medication use more than twice per week, significant exercise limitations or nocturnal awakenings, or exacerbations requiring emergency health care or systemic steroids (SS) more than twice per year [2]. Poorly controlled pediatric asthma leads to over 14 million outpatient visits, nearly 2 million emergency department (ED) visits, and over 400,000 hospitalizations each year, with a disproportionate number of exacerbations being among socioeconomically disadvantaged populations [3,4]. The estimated annual economic impact each year includes over US $50 billion in direct costs, with an additional US $5.9 billion in indirect costs due to lost earnings for caregivers [5-9].

Adolescents are particularly at high risk for poorly controlled asthma and worse outcomes compared to their younger peers. These risks are due to several factors, including poor self-management skills, medication nonadherence, failure to recognize asthma symptoms, and belief that asthma medications are not beneficial [10-16]. Factors leading to nonadherence among adolescents are lack of motivation, social or academic obligations, inadequate parental support, and forgetfulness [17,18]. Despite these risks, adolescents are expected and encouraged by their parents and health care providers to assume more responsibility in the daily management of their chronic illness [19]. Interventions are needed to engage adolescents at high risk for exacerbations. These interventions should aim to improve asthma self-management and enhance adolescents’ knowledge and skills to take appropriate actions for daily asthma maintenance and in response to acute symptoms.

Personalized asthma action plans (AAPs) serve as tools to assist patients with self-management, and current best practices recommend that every patient with asthma have an AAP [2]. AAPs provide patients with instructions for day-to-day asthma monitoring and medication management, as well as provide instructive steps to take during periods of acute or worsening symptoms. Standard AAPs are written using a traffic light design, with green, yellow, and red zones to reflect symptoms. The green zone depicts times when the patient’s asthma is asymptomatic, and the AAP provides instructions for daily controller medications. The yellow and red zones depict times of mild and moderate-to-severe increasing symptoms, respectively. Instructions in the yellow and red zones typically include stepwise guidance to reduce symptoms, such as use and dose of rescue medication and further instructions on when to seek medical care [2]. Although there is strong evidence demonstrating significantly better outcomes, including reduced health care use when personalized AAPs are used, there are significant barriers to adequate use of paper-based AAPs [20].

To address barriers with written or paper-based AAPs, we developed and conducted feasibility testing of a prototype of the Pulmonary Education and Asthma Knowledge Mobile Asthma Action Plan (PEAKmAAP) among 20 adolescents with persistent asthma over an 8-week study period [21,22]. We demonstrated the feasibility of implementing a smartphone-based mobile health (mHealth) intervention for adolescents, and findings suggested that this innovative tool could result in significant improvements in asthma self-efficacy, as well as clinically significant improvements in ACT scores [23]. among at-risk adolescents. Next, we conducted a pilot randomized controlled trial (RCT) test among 34 adolescents over 6 months to compare the use of the PEAKmAAP prototype in paper-based AAPs [24]. Participant satisfaction was high, with 100% of adolescents stating they would recommend PEAKmAAP to a friend. Further, participants with uncontrolled asthma benefited from use of PEAKmAAP, as suggested by clinically important improvements in Asthma Control Test (ACT) scores (defined as a ≥3-point increase in the ACT score) [23] when compared to participants using paper-based AAPs [24]. Herein, we present the protocol of a full scale RCT aimed to examine the effectiveness of PEAKmAAP, a personalized, interactive smartphone app, among adolescents with uncontrolled asthma. We hypothesize that PEAKmAAP participants will experience reduced asthma morbidity compared to the enhanced usual care (eUC) participants and that sharing PEAKmAAP data with primary care providers (PCPs) will be associated with sustained asthma control over the 12-month intervention period.

## Methods

### Study Design

**Figure 1:**
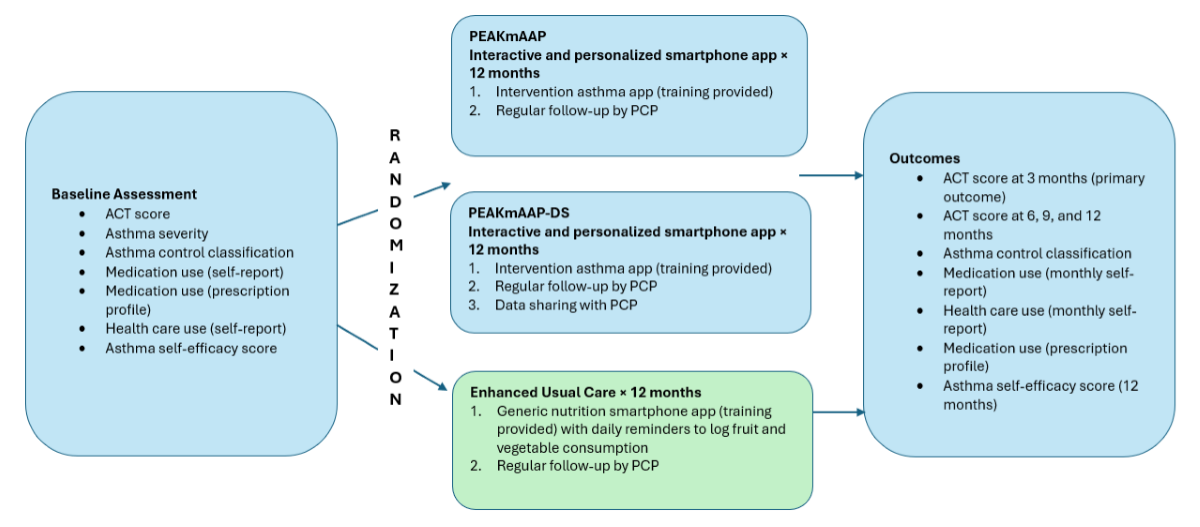
Adolescents (age 12-20 years) with uncontrolled persistent asthma will be randomized to 1 of 3 groups: (1) PEAKmAAP alone, (2) PEAKmAAP-DS, and (3) eUC (using a generic nutrition app, Nutrimap, to log daily fruit and vegetable consumption). Outcomes will be assessed at 3, 6, 9, and 12 months. ACT: Asthma Control Test; PCP: primary care provider; PEAKmAAP: Pulmonary Education and Asthma Knowledge Mobile Asthma Action Plan; PEAKmAAP-DS: Pulmonary Education and Asthma Knowledge Mobile Asthma Action Plan with data sharing.

The study is a 3-arm RCT with adolescents with uncontrolled asthma (Figure 1). The study will be conducted at the Arkansas Children’s Hospital (ACH), with children receiving asthma care at ACH primary and specialty care clinics. A sample of 370 adolescents (age 12-20 years) and their caregivers will be enrolled. Participants will be randomized 1:1:1 to 1 of 3 groups: (1) a group using PEAKmAAP alone, (2) a group using PEAKmAAP with data sharing (PEAKmAAP-DS), and (3) an eUC group using an app with nonasthma daily reminders. Randomization will be stratified according to 2 strata based on asthma severity status (mild persistent and moderate-to-severe persistent) in order to minimize differences in baseline severity that may potentially bias treatment effects. A 1:1:1 permuted block randomization scheme with varying block sizes will be independently generated by our biostatisticians using nQuery Advisor 7.0 within each stratum to conceal allocation. The randomization list will be kept independent of the research staff to ensure allocation concealment.

The study is registered at ClinicalTrials.gov (NCT03842033). SPIRIT (Standard Protocol Items: Recommendations for Interventional Trials) reporting guidelines were used to create this manuscript (Multimedia Appendix 1) [25]. Adherence of this report to the SPIRIT guidelines was supported by a tool developed under R01LM014079.

The inclusion criteria are as follows: (1) age ≥12 and ≤20 years; (2) physician-diagnosed persistent asthma using national guidelines criteria [2]; (3) uncontrolled symptoms, as measured by an ACT score of ≤19 [26,27]; (4) prescription of a preventative (controller) asthma medication in the 6 months prior to enrollment; (5) access to a smartphone compatible with the PEAKmAAP app; and (6) access to the internet.

The exclusion criteria are as follows: (1) any significant underlying respiratory diseases other than asthma, (2) current smoker, (3) moderate-to-severe developmental delay that interferes with the participant’s ability to self-monitor their asthma, and (4) participation in any asthma clinical trials in the 6 months prior to enrollment.

### Ethical Considerations

The study protocol was reviewed and approved by the Institutional Review Board (IRB) of the University of Arkansas for Medical Sciences (UAMS; protocol #206110). All protocol modifications will be approved by the IRB.

Informed consent and assent will be obtained from caregivers and participants, respectively. All participants will be provided with an opportunity to opt out of any study procedures or withdraw from the study at any time.

The principal investigator (PI) will carefully monitor study procedures to protect the safety of research participants, the quality of the data, and the integrity of the study. The PI and unblinded study staff will have access to the data throughout the project. Each participant will be assigned a unique ID. Only study staff will have access to the IDs and information that identify the participants in this study. The electronic and hard-copy files with the participant IDs and study information will be kept separate and stored in a locked office at Arkansas Children’s Research Institute (ACRI) and will be accessible to research personnel only. Only deidentified data will be used for data analysis. At the end of the study, the records will be destroyed in compliance with ACRI and UAMS research policies and guidelines. Electronic records will be stored on the ACRI/UAMS servers, which are password-protected.

To ensure confidentiality, several features have been included in the PEAKmAAP and Nutrimap apps to protect participants’ health information in the event their peers look at their phones:

- The app is password-protected; therefore, only the participants and their caregivers will have access to the mobile and web-based features of the app, including the participants’ protected health information (PHI).
- The app will time-out after 5 minutes of inactivity. The participant’s unique password will have to be re-entered after 10 minutes of inactivity. Further, if the participant switches to a different app, they will be required to re-enter their password when they relaunch the app.
- In the event the participant purchases or acquires a new phone, the app will need to be downloaded to the new phone. The participant will be asked to notify the research personnel when they acquire a new phone. At the time of consent, the participant will receive step-by-step instructions on how to download the app to their phone. An additional link containing the instructions can also be sent to the participant, as requested, at a future date.
- Data entered into the app will not be not permanently stored on the mobile device. Data will only be permanently stored on a UAMS Health Insurance Portability and Accountability Act (HIPAA)-compliant server. If the participant enters data while not connected to a Wi-Fi network, the data will be temporarily stored on the participant’s mobile device and automatically transferred to the HIPAA-compliant server the next time the participant’s device is in an area with a Wi-Fi network. At the end of the 12-month study period, the research personnel will disable the app. The research team will notify the participant that the app is disabled and that it may be deleted from their phone.

Each participant will receive a US $50 incentive for completing the baseline and 3-month visits and a US $100 incentive for completion of the 12-month visit. Participants will also receive US $15 for completion of each monthly survey and up to US $15 each month for daily logging on PEAKmAAP or Nutrimap (US $0.75 per day up to a maximum of US $15 per month). Participants completing all study visits and earning the maximum amount for daily logging will receive a total of US $560 for 12-month study participation. Participants will be offered transportation vouchers, if needed, to travel to the ACH for any in-person visits at baseline, 3 months, or 12 months.

### Community Advisory Board

Prior to the study’s start, the research team convened a Community Advisory Board (CAB) consisting of adolescents with asthma and caregivers of adolescents with asthma. The CAB partnered with the research team and met monthly over a 12-month period to review the findings of the pilot studies [21,22,24] and provide input in an iterative user-centered design process. The CAB conducted beta testing with the PEAKmAAP prototype and provided feedback to the research team regarding functionality and advised the team on the final app’s design prior to participant recruitment. The CAB also provided content ideas and feedback on educational videos and asthma tips sent via push notifications from the PEAKmAAP app.

### Conceptual Models Informing the Intervention’s Design

The intervention is based on the Chronic Care Model (CCM) [28]. This model was chosen because it relates specifically to chronic illness management, and we developed our intervention to include components that foster improved self-management, integrate guideline-based principals into daily life, and support productive interactions between patients and health care teams [29]. CCM components have been used in many interventions, with improved outcomes among patients with asthma [30,31]. Including multiple components of the CCM is associated with stronger effects on medication adherence outcomes among patients with asthma [32]. Our intervention incorporates 3 components of the CCM (Table 1) based on input from the CAB in a method that is convenient, acceptable, and interactive with adolescents on a daily basis.

**Table 1:** PEAKmAAP’s^a^ design and app features informed by the 3 components of the CCM^b^.

CCM component	App features
Self-management	Daily cue to self-monitor with immediate feedback Daily cue to take controller medication(s) Weekly mobile asthma education
Decision support	Guidelines-based algorithms to prompt appropriate care during worsening symptoms
Clinical information system	HIPAA^c^-compliant web portal for 24/7 AAP^d^ access Medication and prescription refill reminders Data sharing with PCPs^e^ (PEAKmAAP-DS^f^ group only) National Weather Service data

^a^PEAKmAAP: Pulmonary Education and Asthma Knowledge Mobile Asthma Action Plan.

^b^CCM: Chronic Care Model.

^c^HIPAA: Health Insurance Portability and Accountability Act.

^d^AAP: asthma action plan.

^e^PCP: primary care provider.

^f^PEAKmAAP-DS: Pulmonary Education and Asthma Knowledge Mobile Asthma Action Plan with data sharing.

**Figure 2:**
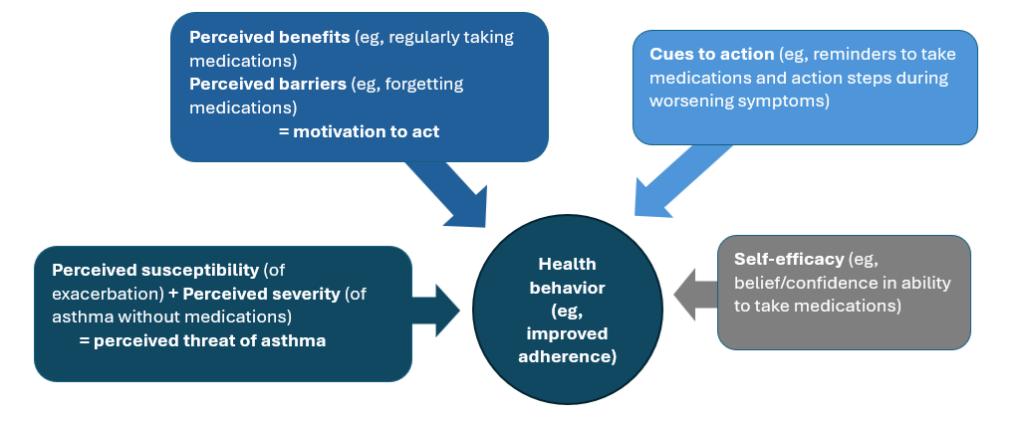
PEAKmAAP’s incorporation of the HBM to affect behavior change and to improve medication adherence. HBM: Health Belief Model.

Medication nonadherence is the primary barrier to asthma control among adolescents [10,11]. Medication adherence is a complex behavior, and behavior change is most likely to occur when a patient is motivated and feels confident in their ability to change [33]. The latter notion refers to self-efficacy. The intervention incorporates the Health Belief Model (HBM) to affect behavior change, for example, increase self-efficacy and increase medication adherence (Figure 2). The HBM is a commonly used explanatory theory of health behavior that has been applied to adolescents [34,35] and specifically to beliefs about medication adherence and asthma management among those with asthma [11].

### Participants

All enrolled participants will receive care from their usual primary care or specialty care provider at the ACH. At baseline, all participants will receive a printed paper copy of their personalized AAP from the electronic health record (EHR), as prescribed by their health care provider. Trained research personnel will provide each participant with training on how to use their AAP according to their health care provider’s instructions. All participants will use a smartphone app during the 12-month enrollment (details described later).

#### Enhanced Usual Care Participants

The eUC participants will receive a nutrition app, Nutrimap, designed by the study team for attention control and daily interactions with a mobile app. Nutrimap will send eUC participants a daily push notification to remind them to eat fruits and vegetables. The application will allow eUC participants to keep a diary of how many servings of fruits and vegetables they consume each day (24-hour recall). Via the Nutrimap app, eUC participants can access the US Department of Agriculture’s (USDA) MyPlate guide [36] to find healthy eating tips, learn how to measure serving sizes, and read about other general nutrition information. The eUC participants will not receive any education or reminders related to asthma from the Nutrimap app. Thus, the eUC participants will still receive their usual asthma care from their asthma doctor or PCP, with the addition of an attention placebo that is not predicted to impact asthma control.

#### PEAKmAAP and PEAKmAAP-DS Participants

Participants in the PEAKmAAP and PEAKmAAP-DS groups will access the smartphone-based, interactive version of their personalized AAP, as prescribed by their asthma care provider. At enrollment, adolescents will receive push notifications via PEAKmAAP, with links to educational videos 2-3 times per week for 6 weeks after the baseline visit in order to reinforce key asthma education topics (Table 2). The videos are informed by CAB members’ input. The videos will remain accessible to participants on the PEAKmAAP app dashboard throughout the 12-month intervention. In addition, participants will be able to log daily controller and rescue medication doses (optional). The app will provide reminders for daily controller medication doses and monthly prescription refills. PEAKmAAP will prompt the adolescents to self-monitor asthma daily and send them a reminder to enter their symptom profile or peak flow (PF) value daily during the time that they experience worsening asthma symptoms. Innovative features of PEAKmAAP and how these features deliver CCM components and HBM concepts are described in detail later. In addition to the same features of PEAKmAAP, the PCPs of PEAKmAAP-DS participants will receive monthly reports with app-generated data regarding the number of days per month they input symptoms or PF values in the green, yellow, or red zone, as well as results of a monthly mobile survey regarding health care use.

**Table 2:** Weekly PEAKmAAP^a^ educational content.

Intervention week (number of videos, n) and themes		Specific topics
**Week 1 (n=2)**		
	Asthma diagnosis	What is asthma?
	Assessment and monitoring	Monitoring asthma and goals of asthma therapy to reduce impairment
**Week 2 (n=3)**		
	Self-management	Asthma emergencies and what to do during an asthma attack Basic facts about asthma (symptoms, causes, and airflow obstruction) Managing asthma in the long term
**Week 3 (n=3)**		
	Treatment (part 1)	What is an AAP^b^? Difference between long-term controller and quick-relief medications Types of long-term controller medications
**Week 4 (n=2)**		
	Treatment (part 2)	How to use a metered dose inhaler, spacer, and nebulizer How to use a discus inhaler (Diskus) and a twist inhaler (Turbuhaler)
**Week 5 (n=3)**		
	Triggers	What are asthma triggers and allergic thresholds? Examples of indoor and outdoor asthma triggers How can I reduce my exposure to triggers?
**Week 6 (n=2)**		
	Allergy testing	What testing can I get for allergies?
	Goals of asthma management	Reducing impairment due to asthma

^a^PEAKmAAP: Pulmonary Education and Asthma Knowledge Mobile Asthma Action Plan.

^b^AAP: asthma action plan.

### PEAKmAAP Application Features

#### HIPAA-Compliant Web-Based Portals for the Study Team and Participants/Caregivers

Incorporating the *clinical information system* component of the CCM, PEAKmAAP includes HIPAA-compliant, password-protected portals to ensure data accessibility for both participants and the study team (Table 3). A web-based portal will be available to participants/caregivers to allow 24/7 access to them over the internet. This feature will also allow for participant data entry in the event of a lost, stolen, or damaged phone or lost mobile service. The study team’s portal will allow research personnel to create and sync participants’ personalized AAP instructions onto the participants’ PEAKmAAP app and to set preferences for notifications and reminders.

**Table 3:** Features of PEAKmAAP^a^ and incorporation of CCM^b^ components and HBM^c^ concepts.

PEAKmAAP features	Purpose	CCM component	HBM concept
HIPAA^d^-compliant web portal	Provides 24/7 access to medical instructions in the case of a lost phone or lost phone service	Clinical information system	N/A^e^
Asthma self-monitoring	Reminds patients to monitor asthma symptoms or PF^f^ daily	Self-management	Cue to action, self-efficacy
Personalized treatment plans	Provides a guideline-based algorithm to take controller or rescue medication	Decision support	Perceived severity, perceived barrier
Medication reminders	Provides daily medication and monthly refill reminders	Clinical information system, self-management	Cue to action
Mobile educational tips	Delivers educational tips and other resources	Self-management	Perceived severity and susceptibility, perceived benefits and barriers, self-efficacy
National Weather Service data	Provides data of environmental changes that could impact asthma	Clinical information system	N/A
PCP^g^ data sharing (PEAKmAAP-DS^h^)	Generates data for monthly PCP reports	Clinical information system	N/A

^a^PEAKmAAP: Pulmonary Education and Asthma Knowledge Mobile Asthma Action Plan.

^b^CCM: Chronic Care Model.

^c^HBM: Health Belief Model.

^d^HIPAA: Health Insurance Portability and Accountability Act.

^e^N/A: not applicable.

^f^PF: peak flow.

^g^PCP: primary care provider.

^h^PEAKmAAP-DS: Pulmonary Education and Asthma Knowledge Mobile Asthma Action Plan with data sharing.

#### Asthma Self-Monitoring and Personalized Treatment Plans

Incorporating the *self-management* component of the CCM, PEAKmAAP and PEAKmAAP-DS participants will be prompted to complete an asthma symptom checklist or enter a PF rate daily. They will also access PEAKmAAP to enter symptoms or the PF during worsening symptoms, which is an overt cue to action as part of the HBM. Adolescents may be recommended by their asthma care provider or have a personal preference to record either symptoms or the PF. The app is programmed to navigate to the appropriate personalized asthma zone when *either* symptoms or the PF are entered; participants *are not* required to enter both. Once data are entered (PF or symptoms), PEAKmAAP will automatically navigate to a new color-coded green, yellow, or red landing page to provide real-time instructions that correspond to the participant’s personalized green, yellow, or red zone of their AAP. The instructions assist the adolescent in determining when to take controller medications, what the rescue medication dose and frequency are, and when to contact medical personnel according to instructions prescribed by their asthma care provider.

Personalized treatment zones will alert the adolescent to a more accurate level of perceived severity based on their entered data. This will also incorporate the *decision support* component of the CCM. In addition to prompting appropriate action, providing personalized information will address the perceived barrier of not knowing their treatment options by eliminating guesswork, a process that builds self-efficacy, which is key to behavior change. If participants navigate to the green zone, instructions for daily controller medications will appear. If they navigate to the yellow or red zone, instructions for rescue medication dosing and frequency will appear (cue to action), in addition to instructions to take daily controller medications. The PEAKmAAP app will provide several opportunities for *corrective actions* to be taken in the event of acute symptoms:

- Follow-up messages will prompt the adolescent to reassess asthma 20 minutes after any yellow or red zone recording. If the adolescent fails to respond, a second prompt will be sent 20 minutes later.
- If the adolescent enters severe symptoms (eg, lips turning blue, tightness in the throat), the app will automatically navigate to a shortcut prompting them to “Call 911/EMS” or seek emergency care.

In alignment with asthma guidelines [2] and instructions provided on paper-based AAPs, the PEAKmAAP app’s design includes additional cues to action by defining parameters for prolonged or severe exacerbations. A prolonged exacerbation is defined as recording of yellow zone readings on 2 or more consecutive days *or* failing to indicate an improvement (eg, answers no or does not respond to the question, Are you feeling better?) within 48 hours of a yellow zone alert. A severe exacerbation is defined as ≥2 consecutive red zone readings in a ≤1-hour period *or* failing to indicate improvement within 1 hour of a red zone alert. In the event of a prolonged or severe exacerbation, the adolescent will be prompted via PEAKmAAP to contact their doctor. For prolonged or severe exacerbations, an alert will be sent to the participant and study team web portals.

PEAKmAAP is not intended or designed to replace participants’ medical care. Participants will be counseled during the informed consent/assent process regarding the importance of seeking appropriate medical care. Caregivers and participants will be advised to seek appropriate medical care, including the participants’ asthma care providers, emergency care, or the ACH Afterhours Nurse Line for worsening or uncontrolled symptoms. As a safety precaution, trained study personnel will monitor the study team’s web portal during regular business hours and contact the participant’s caregiver within 1 business day of any prolonged or severe exacerbation alert to ensure that appropriate medical attention is sought.

#### Customizable Medication Reminders

Incorporating the *clinical information system* component of the CCM, PEAKmAAP and PEAKmAAP-DS participants will receive daily messages (cue to action) to take controller medication doses. Via push notifications, participants will receive a prompt—“Don’t forget to take your ___ (participant’s controller)!”—once or twice daily as per their personalized AAPs. Monthly prescription refill reminders (cue to action) will be sent via push notifications for all controller medications. The delivery time of the messages and reminders will be customized based on the participants’ preferences (eg, before school on school days, midmorning on weekends, etc).

#### Mobile Educational Tips and Resources

The PEAKmAAP app incorporates multiple media types (as recommended by the CAB), including videos (Table 2), text, and pictures to deliver education. Asthma education tips will be delivered via push notifications (to address perceived severity and perceived susceptibility of asthma exacerbation). Weekly text messages will deliver short tips to reinforce asthma concepts, such as medication adherence, inhaler/spacer technique, knowing the difference between rescue and controller medications, and recognizing asthma symptoms. In addition to receiving these educational tools via push notifications, participants will have access to all educational content through an “Asthma Tips” shortcut on PEAKmAAP’s home screen.

#### National Weather Service Data

Participants will have access to real-time location-specific weather data from the PEAKmAAP home screen [37]. Weather details (weekly forecast predictions of temperatures in Fahrenheit, precipitation, humidity, pollen count, and ozone level) will be provided to help adolescents be more aware of environmental changes that could trigger asthma symptoms.

#### Data Sharing

Data generated by PEAKmAAP and mobile surveys (detailed later) will be shared with the asthma care providers of PEAKmAAP-DS participants monthly. The monthly report will include (1) results of monthly mobile surveys documenting rescue medication use, acute health care visits, and prednisone bursts and (2) PEAKmAAP-generated data regarding prolonged or severe exacerbations (described earlier).

### Summary of Study Visits

#### Screening

Potentially eligible participants were recruited from primary care and specialty clinics at the ACH through direct patient contact and advertising. In addition to direct patient contact in clinics, the research team has received IRB approval to identify potentially eligible subjects through the EHR by prescreening for age and asthma diagnosis eligibility criteria prior to clinic appointments at primary care and specialty clinics. Potentially eligible participants were called prior to their clinic visit to allow research personnel to introduce the study and to ask permission to discuss the study in detail at their upcoming clinic visit. Because ACH patients receive care in other settings, such as the ED and other outpatient clinics, or may be registered in institutional research participant registries, participants were also recruited from these locations.

#### Baseline Visit

Research personnel obtained informed consent and assent from caregivers and adolescent participants, respectively, prior to any study procedures. At the initial visit, baseline data collection included lung function testing, frequency of asthma symptoms, activity limitations, health care use, asthma self-efficacy, and assessment of the dose and frequency of asthma rescue and controller medication. After these baseline data were obtained, participants were randomized to 1 of the 3 arms of the study. After randomization, PEAKmAAP and PEAKmAAP-DS participants received detailed instructions for use of the PEAKmAAP app, including an instructional video to explain all the features of the app, while the eUC participants received detailed instructions for the use of Nutrimap (Table 4).

**Table 4:** Summary of study visit, outcome measures, and survey documents.

Measure	Screening prerandomization	Baseline prerandomization	3 months	6 months	9 months	12 months
Eligibility	X^a^	—^b^	—	—	—	—
ACT^c^ survey	X	Screening ACT score	X^d^	X	X	X^e^
Asthma severity classification	—	X	—	—	—	—
CASI^f^ score	—	X	X	—	—	X^e^
Lung function test with fraction of FeNO^g^	—	X	X^e^	—	—	X^e^
Child Self-Efficacy Survey	—	X	X^e^	—	—	X^e^
Asthma control classification (derived from the ACT survey)	—	X	X^e^	X	X	X^e^
Medication adherence (prescription profile for prior 12 months)	—	X^h^	—	—	—	X^h^
System Usability Scale (SUS)	—	—	—	—	—	X
Medication use (monthly self-report)	—	X	Monthly	Monthly	Monthly	Monthly
Health care use (monthly self-report)	—	X	Monthly	Monthly	Monthly	Monthly

^a^X: applicable.

^b^Not applicable.

^c^ACT: Asthma Control Test.

^d^Primary outcome.

^e^Outcome obtained by blinded research personnel at an in-person visit (all others via a mobile survey).

^f^CASI: Composite Asthma Severity Index.

^g^FeNO: fractional exhaled nitric oxide.

^h^Profile requested directly from insurer, pharmacy data, or medical record.

#### Monthly Surveys

All participants receive a link to a survey each month via push notifications from their mobile app (PEAKmAAP or Nutrimap). The monthly survey asks participants to self-report rescue medication use in the past 2 weeks due to wheezing, coughing, or trouble breathing. The survey also asks participants to record exacerbations resulting in hospitalization, ED or sick visits, or prednisone use in the previous 4 weeks. These data, along with app-generated data on the frequency of green, yellow, and red zone days, will be used to generate the PCP report for PEAKmAAP-DS participants.

#### Three- and Twelve-Month Visits

Three and twelve months after enrollment, an in-person visit will be conducted with all participants to obtain lung function testing results. Blinded research personnel will survey the participants to complete the ACT and self-efficacy questionnaire and to obtain a detailed history on asthma symptoms and activity limitations. The 3-month ACT score is the primary outcome of the study.

#### Six- and Nine-Month Visits

Six and nine months after enrollment, a telephone visit will be conducted to administer the ACT. Research personnel conducting the visit will be blinded to every participant’s treatment arm.

### Summary of Outcome Measures

#### Primary Outcome Measure

The primary outcome measure will be the change in the ACT score of ≤19 (uncontrolled asthma) to >19 (controlled asthma) at 3 months. The ACT is a validated 5-question survey used to assess asthma control in individuals ≥12 years of age. The ACT is used for symptoms monitoring as per guidelines [2], and the ACT score has been identified as an appropriate asthma control composite score in clinical research [26]. The ACT is an efficient, reliable, and validated method of measuring asthma control, with an internal consistency reliability of 0.84 [27]. Mobile and web-based versions of the ACT have been validated [38,39], and we have successfully used the ACT in our pilot studies [21,40].

#### Secondary Outcome Measures

##### ACT Survey to Assess Secondary Outcomes

Recognizing that ACT scores may improve but remain ≤19 for some participants with poorly controlled asthma, we will also assess for change in the ACT score that is considered clinically important (as indicated by a ≥3-point increase) [23]. The ACT score will be assessed via a mobile survey to measure for sustained improvement throughout the study. We hypothesize that PCP data sharing will facilitate asthma control by alerting the PCP when asthma worsens or fails to improve. The impact of PCP data sharing on the ACT score will be measured to assess whether data sharing has added any benefit to the PEAKmAAP group.

##### Composite Asthma Severity Index

The Composite Asthma Severity Index (CASI), a comprehensive scale reflecting impairment, future risk, and the level of treatment needed to maintain control, has been developed to align with the new concept recommended in *Expert Panel Report 3: Guidelines for the Diagnosis and Management of Asthma* (EPR-3) [2]. The integral CASI instrument has 5 domains: day symptoms and albuterol use, night symptoms and albuterol use, controller medication, lung function measures, and exacerbations. CASI is considered a better discriminator of treatment response than a symptom-based outcome. Thus, as an alternative to the ACT score, CASI will be also used to quantify asthma severity and prospectively gauge asthma control in the proposed study.

##### Medication Use

Self-reported rescue medication use will be assessed via a mobile survey. Participants will be asked, “How many days/nights in the past 2 weeks did you use ___ (rescue medication name) because you were wheezing, coughing, or having trouble breathing?” These data will be collected for all participants and will be used to generate a PCP report for PEAKmAAP-DS participants.

##### Health Care Use

Self-reported health care use will be assessed monthly via a mobile survey. Participants will record exacerbations resulting in hospitalization, ED or sick visits, or prednisone use. These data will be collected for all participants and will be used to generate a PCP report for PEAKmAAP-DS participants.

##### Lung Function and Airway Inflammation

Spirometry is the test most used for the assessment of the risk for adverse events (AEs) and impairment in asthma [2]. The forced expiratory volume in the first second (FEV1) expressed as a percentage predictive of the reference population value or as a proportion of the forced vital capacity (FEV1/FVC) will be assessed. Fractional exhaled nitric oxide (FeNO) is a quantitative, noninvasive measure of airway inflammation that predicts responsiveness and adherence to inhaled corticosteroids (ICS) [41-43]. We will measure lung function and FeNO at baseline, 3 months, and 12 months for all participants.

##### Child Self-Efficacy Survey.

The Child Self-Efficacy Survey is a 9-item validated questionnaire and is designed to measure a child’s self-efficacy with regard to attack prevention and attack management [44]. The participants will be required to select 1 of 4 responses ranging from “none of the time” (0 points) to “all of the time” (3 points). The survey will be administered at baseline, 3 months, and at the end of the intervention for all participants. The reliability of the survey (Cronbach α) is .75.

##### Asthma Control Classification

Asthma guidelines categorize asthma control for individuals ≥12 years old into 3 categories: well-controlled (WC), not well-controlled (NWC), or very poorly controlled (VPC) [2]. Asthma control is defined by the degree to which asthma manifestations are minimized by therapeutic intervention [2]. Components of asthma control include measures of daytime symptoms, nighttime awakenings, activity limits, rescue medication use, and exacerbations requiring oral systemic corticosteroids. An assessment of asthma control will be performed during the eligibility screening; at the in-person, structured interviews at baseline, 3 months, and 12 months; and via a mobile survey at 6 and 9 months for all participants.

##### Medication Adherence

Using prescription refill data, we will compare adherence prior to enrollment with adherence during the study period. We have successfully attained prescription refill data in our previous studies. Participants will provide written release of information during the consent interview. In addition, these data are frequently requested by providers as a component of usual patient care in Primary Care Medical Home (PCMH) clinics. Adherence will be defined as receiving ≥3 controller medication refills/6 months or achieving an asthma medication ratio (AMR) of ≥0.5 based on national asthma guidelines [2]. The AMR will be calculated as the ratio of the number of controller medications dispensed during a 6-month period divided by the sum of controller plus rescue medications dispensed during the 6-month period. An AMR of ≥0.5 has been associated with improved asthma outcomes [45]. The proportion of days covered (PDC) will also be assessed and compared between groups from prescription refill data. This measurement is defined by the number of days in the period covered divided by the number of days in the total period and is suitable for medication regimens that require multiple medications, such as asthma. Prescription refill data will be captured from the participants’ medical record dispense report via Magellan (a Medicaid vendor) or via a private pharmacy. We may also contact the participants’ local pharmacy used to obtain the pharmacy profile.

##### Medication Changes

To monitor for step-up or step-down in prescribed controller medications, medication changes will be assessed at each in-person visit at 3 and 12 months, by medical record review, and by prescription refill data. Participants will be advised to notify study staff of any medication changes.

### Data Collection and Management

We will use the Research Electronic Data Capture (REDCap) program. Data from the study will be checked for errors, inconsistencies, and missing data. We will examine the data graphically using scatterplots for continuous outcomes and frequency tables for categorical data. Unresolved data anomalies will be sent to the primary investigator for verification. Obvious outliers due to data entry errors will be corrected before data analysis begins. Different data sources will be linked using a unique identifier assigned to each participant.

### Adverse Event Reporting

All subjects will be monitored for AEs related to study procedures. Study investigators acknowledge that participants may experience asthma symptoms or exacerbations throughout the course of the study; however, exacerbations are expected due to the nature of the disease and not due to study-related procedures. Due to an intrinsic exacerbation risk to all patients with asthma, we will advise participants to continue to use medications as prescribed by their PCP and to keep their regularly scheduled visits. The research team will also conduct research review meetings on a weekly basis to monitor the progress of the study, ensure participant safety, discuss any unanticipated problems, ensure treatment integrity, and ensure that recruitment and study goals are being met, as well as solve any issues pertaining to enrollment, recruitment, or completion of study procedures. A Safety Monitoring Committee (SMC) will independently monitor participant safety. The SMC, consisting of a biostatistician and an independent clinical research investigator, will meet no less than once a year and as needed to discuss any potential safety concerns related to the research. All participants will be screened for AEs and serious adverse events (SAEs) at each study visit. They will be asked about asthma- and non–asthma-related health issues. All clinically significant AEs will be brought to the attention of the PI, who will categorize the AEs as “expected” or “unexpected” and as “likely” or “unlikely” to be related to the study. The PI will monitor all related AEs and SAEs. All AEs and SAEs will be entered into the study database and, as required, reported to the University of Arkansas for Medical Sciences Institutional Review Board, the SMC, or the National Institutes of Health (NIH). Because the interventions are adjuncts to ongoing clinical care and all participants are encouraged to continue to engage with their PCP, no formal ancillary or posttrial care is planned. Similarly, given the low risk of the interventions and continued PCP care, there are no criteria for discontinuing or modifying allocated interventions beyond a participant’s personal decision to discontinue participation in the study.

### Statistical Analysis

Primary models using the intent-to-treat (ITT) principle will be performed for all primary and secondary outcomes. All subjects will be considered part of their assigned treatment groups irrespective of their adherence to treatment, and all randomized subjects will be included in the analysis. Secondary models using a per protocol (PP) population including only participants who adhered to the protocol will be conducted. As appropriate, models will be adjusted for the baseline value of the outcome, with other baseline confounding or covariate variables included. Secondary subgroup analyses will stratify by baseline asthma control classification (poorly controlled vs not well controlled) defined by ACT scores. Holm-Bonferroni correction will be used to control the overall familywise error rate under 5% for analyses with multiple comparisons. Missing data in primary and secondary models will be handled by multiple imputation if “missing at random” is a reasonable assumption. Results will also be reported discarding missing values as a sensitivity analysis.

Using these models, the primary outcome of the proportion of participants with ACT scores of >19 at 3 months will be analyzed using linear models with a logit link function and contrasts to compare the 2 PEAKmAAP groups combined with the eUC group. Secondary analyses of the proportion of ACT scores of >19 at additional timepoints (6, 9, 12 months) will be assessed with contrasts for the group effect (to measure the average effect across the different timepoints) and the group-by-time interaction effect (to measure the change in effect over time) between groups (PEAKmAAP-DS vs PEAKmAAP; PEAKmAAP groups combined vs eUC). Since the ACT score will be measured at 3, 6, 9, and 12 months (all participants will have baseline scores of ≤19), these correlated repeated measures data will be analyzed with a binomial distribution and a logit link function for modeling with an appropriate correlation structure (eg, compound symmetry or first-order autoregressive). Additionally, minimally important improvements in ACT scores (3-point increase) and CASI (1-point decrease) will be similarly modeled as binary outcomes across timepoints, contrasting eUC versus the 2 PEAKmAAP groups combined or PEAKmAAP versus PEAKmAAP-DS. Other secondary outcomes (health care use, rescue medication use, lung function) will be analyzed using similar models for nonnormal data using appropriate distributions and link functions.

Medication adherence rates at 12 months will be compared using a logistic regression model after adjusting for baseline values as a covariate, contrasting eUC against the 2 PEAKmAAP groups. Mean asthma self-efficacy scores at 3 and 12 months, including the subscales for prevention and management, will be compared among the 3 groups using the analysis of covariance model after adjusting for baseline values as a covariate in the model. The proportion of adolescents who receive step-up therapy will be compared between the 2 PEAKmAAP groups, and the 2 PEAKmAAP groups together will be compared with the eUC across different timepoints using a similar logistic model.

No interim analyses are planned, and thus, there is no planned stopping rule for the study.

### Sample Size and Power Calculation

The sample size was determined to detect a clinically meaningful difference between PEAKmAAP and PEAKmAAP-DS groups combined versus the eUC group for the primary outcome measure, which is the proportion of adolescents with ACT scores of >19 at 3 months. We estimate that the proportion of participants with ACT scores of >19 at 3 months will be approximately 15% higher in the PEAKmAAP and PEAKmAAP-DS groups compared to the eUC group based on pilot work [24,40]. Using a generalized estimating equations (GEE) model for binary longitudinal data, we estimated that a sample size of 107 participants in each group will yield at least 80% power to detect the difference mentioned before. After adjusting for a 15% attrition rate, the final sample size needed will be 124 in each group (372 total). The sample size will also have at least 80% power to detect a change from a baseline of 4 between the eUC group and PEAKmAAP and PEAKmAAP-DS groups combined for the asthma self-efficacy score. The SD of the asthma self-efficacy score was determined to be 7 [21,22]. The sample size determination was performed using the GEESIZE macro in SAS Software and PASS [46-48]. All sample size calculations assumed a 2-sided significance level of 5%.

## Results

**Figure 3:**
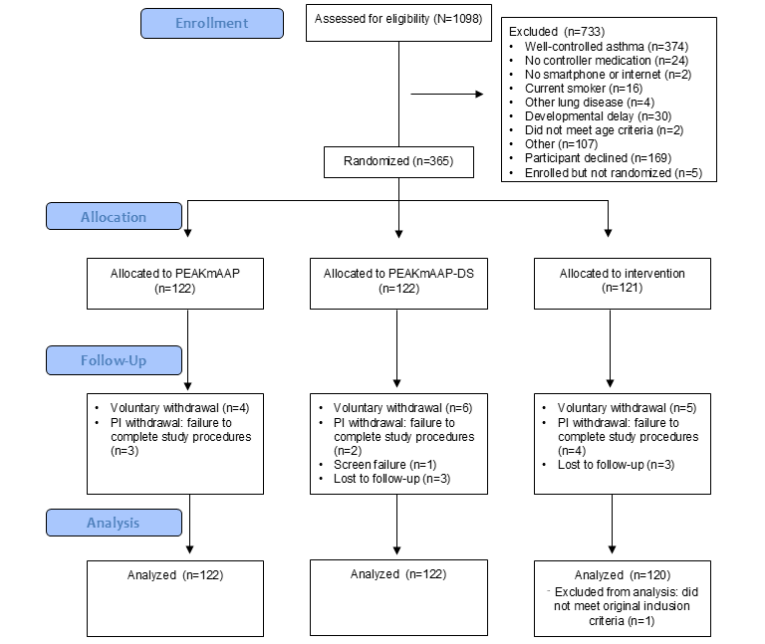
Cumulative screening and enrollment summary. PEAKmAAP: Pulmonary Education and Asthma Knowledge Mobile Asthma Action Plan; PEAKmAAP-DS: Pulmonary Education and Asthma Knowledge Mobile Asthma Action Plan with data sharing; PI: principal investigator.

Recruitment and data collection began in March 2019 and were completed May 2024. Figure 3 depicts the number of participants screened, enrolled, and randomized. Full data analysis began in December 2024.

Peer-reviewed publications are planned, notably the primary outcome manuscript. In addition, publications describing PEAKmAAP app development, a description of population digital access at baseline, process variables describing usability of the app, and other secondary outcomes papers will be considered. Scientific and professional conference abstracts will be or have been submitted. Authorship will be determined based on guidance of the International Committee of Medical Journal Editors.

All analytical code will be made publicly available through the Open Science Framework or in a more tailored repository, as appropriate.

## Discussion

### Summary

Adolescents with asthma are at increased risk for adverse health outcomes related to asthma and are a group traditionally difficult to engage in self-management strategies for chronic illnesses. Smartphone use is prevalent and has been cited as a preferred and convenient method of communication among adolescents. mHealth delivered via smartphones can provide health education, medication tracking and logging, and chronic disease management integration into the daily lives of patients with asthma [49]. The current literature examining the role of mHealth apps for adolescent asthma is limited. Most studies are small pilot trials with a small number of participants or a short study period. We will test PEAKmAAP’s effectiveness among a large cohort of high-risk adolescents with uncontrolled asthma over a 12-month period. Based on the CCM and by virtue of its convenience, ability to provide immediate feedback, and ease of integration into a daily routine, we will examine the PEAKmAAP intervention as a tool to improve asthma self-management, improve medication adherence, and reduce asthma-related morbidity among adolescents at risk for future exacerbations. Our central hypothesis is that the PEAKmAAP and PEAKmAAP-DS groups will have reduced asthma morbidity compared to the eUC group. We further hypothesize that PCP data sharing will facilitate sustained asthma control and that significantly more adolescents in the PEAKmAAP-DS group will have continued asthma control at follow-up visits compared to PEAKmAAP alone and the eUC group. This novel approach has a high impact potential as it may be applicable to other age groups and patients with other chronic medical conditions.

## Appendix

Multimedia Appendix 1 SPIRIT checklist.

## Abbreviations

AAP: asthma action plan
ACH: Arkansas Children’s Hospital
ACRI: Arkansas Children’s Research Institute
ACT: Asthma Control Test
AE: adverse event
AMR: asthma medication ratio
CAB: Community Advisory Board
CCM: Chronic Care Model
ED: emergency department
EHR: electronic health record
eUC: enhanced usual care
FEV: forced expiratory volume in the first second
HBM: Health Belief Model
HIPAA: Health Insurance Portability and Accountability Act
IRB: Institutional Review Board
mAAP: mobile health asthma action plan
mHealth: mobile health
PCP: primary care provider
PEAKmAAP: Pulmonary Education and Asthma Knowledge Mobile Asthma Action Plan
PEAKmAAP-DS: Pulmonary Education and Asthma Knowledge Mobile Asthma Action Plan with data sharing
PF: peak flow
PI: principal investigator
RCT: randomized controlled trial
SAE: serious adverse event
SMC: Safety Monitoring Committee
UAMS: University of Arkansas for Medical Sciences
